# BCOR mutations define a therapeutic vulnerability to DHODH Inhibition in acute myeloid leukemia

**DOI:** 10.1007/s00277-026-06773-z

**Published:** 2026-01-19

**Authors:** Florian Robert, Cherif Badja, Soraya Boushaki, Andrea Degasperi, Yasin Memari, Sophie Momen, Theodoros I. Roumeliotis, Zuza Kozik, Malgorzata Gozdecka, Jyoti Choudhary, George Vassiliou, Gene CC Koh, Serena Nik-Zainal

**Affiliations:** 1 Department of Medical Genetics, Level 6 Addenbrooke’s Treatment Centre, Cambridge Biomedical Research Campus, Box 238, Cambridge , CB2 0QQ UK; 2 Early Cancer Institute, Department of Oncology, Cambridge Biomedical Research Campus, Hutchison Research Centre, Box 197, Cambridge, CB2 0XZ UK; 3 Cambridge Stem Cell Institute, Jeffrey Cheah Biomedical Centre, Puddicombe Way, Cambridge, CB2 0AW UK; 4 Functional Proteomics Group, Institute of Cancer Research, Chester Betty Labs, London, SW3 6JB UK

**Keywords:** Acute myeloid leukemia, Leukemia, BCOR, DHODH, DHODH inhibition, Synthetic lethality, Targeted therapy

## Abstract

Acute Myeloid Leukemia (AML) remains challenging to treat, especially in cases with mutations in the BCL-6 co-repressor (BCOR), which are associated with poor prognosis and chemo-resistance. In this study, we reveal a synthetic lethal interaction between *BCOR* and dihydroorotate dehydrogenase (DHODH). We demonstrate that *BCOR*-deficient cells have a heightened sensitivity to DHODH inhibitors such as brequinar and leflunomide, that are already in clinical use. We confirm that DHODH inhibition selectively induces cell death in BCOR-mutant cells in multiple cellular models, in malignant and non-malignant cells, through chemical and genetic manipulation. Interestingly, we find that the dependency on DHODH does not stem from its role in *de novo* pyrimidine biosynthesis disruption. Rather, DHODH’s role in the electron transport chain, essential for mitigating reactive oxygen species, may be the physiological vulnerability that pushes BCOR-mutant cells toward cell death when DHODH is inhibited. DHODH inhibitors could be repurposed as targeted therapies for BCOR-mutant tumors, offering a promising strategy for precision medicine in AML and other cancers.

**Supplementary Information:**

The online version contains supplementary material available at 10.1007/s00277-026-06773-z.

## Introduction

Acute Myeloid Leukemia (AML) is a heterogeneous hematologic malignancy marked by uncontrolled proliferation of immature myeloid progenitors [1]. Despite molecular advances, prognosis remains poor, particularly in relapsed or therapy-resistant disease [2]. BCL-6 co-repressor (BCOR) mutations occur in ~ 3–6% of AML [3], enriched in RUNX1-mutant or secondary AML following myelodysplastic syndromes [4]. BCOR, a transcriptional co-repressor within the noncanonical PRC1.1 complex [5], regulates gene expression via epigenetic interactions. Most BCOR mutations are loss-of-function, impairing hematopoietic differentiation and driving leukemogenesis [6]. Clinically, BCOR-mutant AML has inferior outcomes (Figure S.1.A), especially when co-mutated with cohesin, RUNX1, or DNMT3A [6, 7], with increased chemoresistance at relapse [7]. Thus, new therapeutic approaches are urgently needed.

Previously, we observed that human induced pluripotent stem cells (hiPSCs) acquired BCOR mutations during culture, despite absence in patient-derived starting material [8]. While probing mutagenesis from dNTP-pool imbalance, we found BCOR-mutant hiPSCs were selectively sensitive to brequinar, a potent inhibitor of dihydroorotate dehydrogenase (DHODH).

DHODH catalyzes the mitochondrial conversion of dihydroorotate to orotate in de novo pyrimidine synthesis [9], essential for uridine, cytidine, and thymidine nucleotide pools. A screen of > 330,000 compounds in a murine AML model also identified DHODH inhibitors as predominant hits [10]. Subsequent studies of brequinar, leflunomide, and teriflunomide support DHODH inhibition as effective and tolerable in AML, T-ALL, and breast cancer [10]. Calls to repurpose brequinar for AML have followed, though a predictive biomarker remains lacking.

Given the aggressiveness of AML, limited therapeutic options, and our serendipitous finding of BCOR-mutant sensitivity to DHODH inhibition, we tested whether BCOR mutations establish a synthetic lethal interaction with DHODH blockade. If validated, BCOR could represent a biomarker to guide selective DHODH inhibitor use in AML.

## Methods

Detailed experimental methods are available as a Supplementary File.

### Cell culture

OCI-AML2/3 were maintained in α-MEM with 20% FCS; MOLM13, HL60, and SKM-1 in RPMI with 10–20% FCS; RPE1 in DMEM/F12 with 10% FCS; and hiPSCs on Vitronectin in Essential E8 medium (all Gibco).

### siRNA transfection and compound treatment

OCI-AML2/3 and RPE1 cells were transfected with siRNAs targeting BCOR, DHODH, or controls (Thermo Fisher) using Lipofectamine 2000. Cells were harvested after 72 h for RNA isolation, viability, or proliferation assays. For pharmacologic studies, cells were treated with brequinar (Cayman Chemical), TP-021, leflunomide, teriflunomide, AG-636, farudodstat, sparfosic acid (MedChemExpress), or 6-azauridine (Thermo Fisher). Viability was assessed by CellTiter-Glo (Promega).

### CRISPR–Cas9 editing

BCOR knockout in OCI-AML3 was achieved using Cas9 RNP nucleofection with sgRNAs (Synthego) and HDR templates (IDT) via a Lonza 4D-Nucleofector.

### RNA sequencing

RNA libraries (PureLink RNA Mini Kit, Thermo Fisher) were sequenced on Illumina NovaSeq (150 bp paired-end). Reads were aligned with STAR, quantified by featureCounts, and analyzed using DESeq2; pathway enrichment was performed with ShinyGO.

### Whole-genome sequencing

Single-cell clones of RPE1 cells from control and brequinar-treated conditions were subjected to whole-genome sequencing (Illumina HiSeq X-Ten, 30×). Variants were called with CaVEMan, and mutational signatures analyzed using the Mutational Signatures framework and signature.tools.lib.

### ROS, ATP, and Fe²⁺ assays

ROS, ATP, and cytoplasmic Fe²⁺ were quantified using commercial detection kits (OZ Biosciences, Promega, Dojindo) and measured on a Pherastar plate reader. Imaging was performed with an EVOS FL Auto 2 microscope.

### Proteomics

Protein extracts were digested, TMT-labeled, fractionated by high-pH reversed-phase chromatography, and analyzed by Orbitrap Lumos mass spectrometry. Spectra were searched against UniProt with Proteome Discoverer, and reporter ion quantification normalized across TMT batches for differential analysis in R.

### Statistical analysis

Statistical tests were performed in GraphPad Prism 10 using two-tailed t tests or one-/two-way ANOVA with post-hoc corrections. *P* < 0.05 was considered significant.

## Results

### BCOR Inhibition sensitises cells to brequinar

**Fig. 1:**
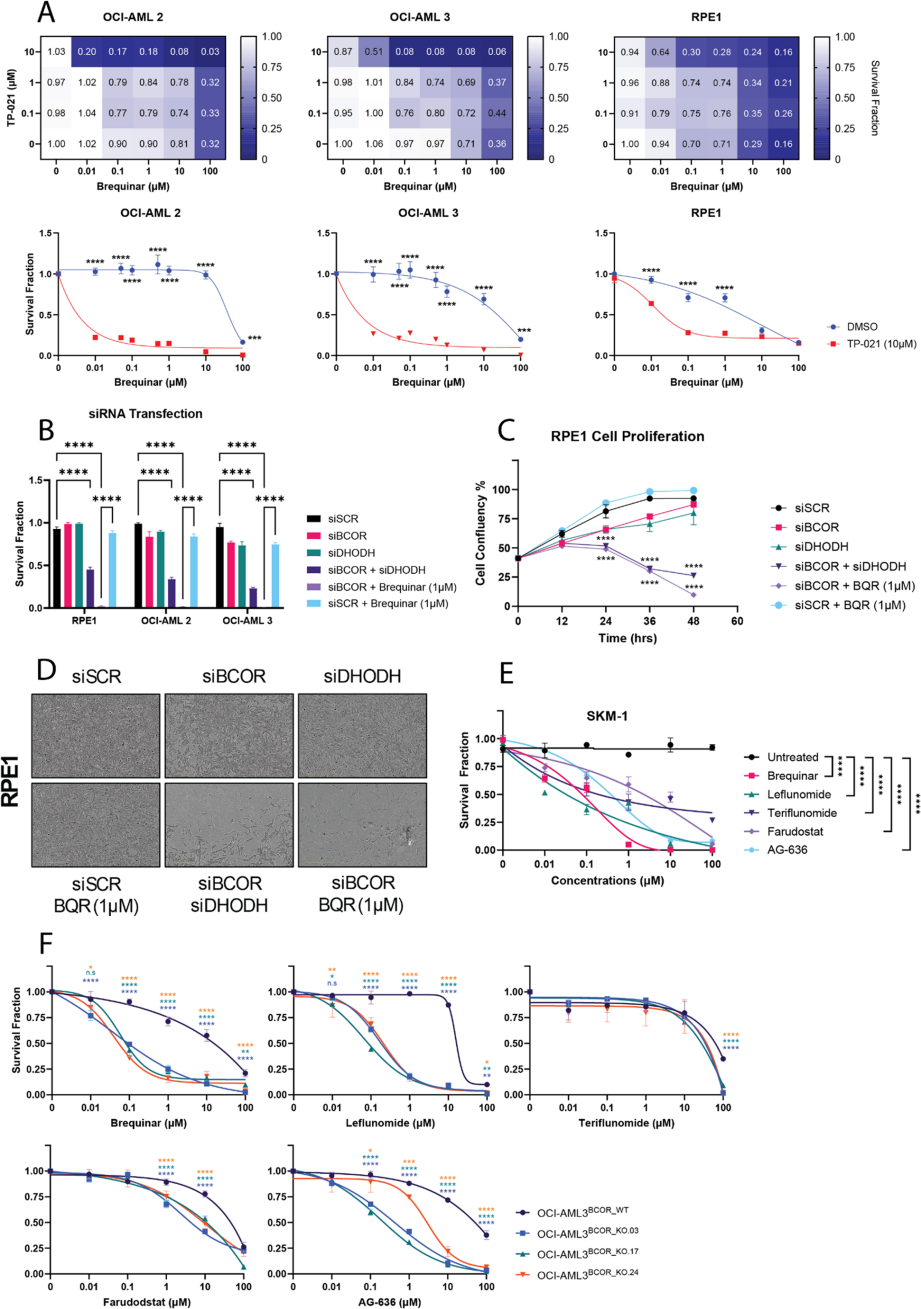
BCOR deficiency sensitizes cells to DHODH Inhibitors. (**A**) Heatmaps representing survival fractions of OCI-AML2, OCI-AML3 and RPE1 treated with increasing doses of TP-021 (BCOR inhibitor) and brequinar, for 6 days (*n* = 3 per line). Survival fractions of OCI-AML2, OCI-AML3 and RPE1 treated with working dose of TP-021 (10 µM) compared to DMSO, with increasing doses of brequinar, for each cell line, for 6 days (two-way ANOVA, *n* = 3; *p* < 0.0001****; *p* < 0.001***). (**B**) Survival fractions of OCI-AML2, OCI-AML3 and RPE1; transfected with siRNA and ± treated with brequinar 1 µM (two-way ANOVA, *n* = 3; *p* < 0.0001****). (**C**) Cell proliferation assay of RPE1 transfected with siRNA, ± treated with brequinar 1 µM (two-way ANOVA, *n* = 2; *p* < 0.0001****) with (**D**) associated pictures taken at 48 h (basic analyzer – Incucyte, 10x magnification). (E) SKM-1 (BCOR-mutated AML) was treated with increasing doses of DHODH inhibitors for 6 days (two-way ANOVA, *n* = 3; *p* < 0.0001****). (**F**) OCI-AML3^BCOR_WT^, OCI-AML3^BCOR_KO.03^, OCI-AML3^BCOR_KO.17^ and OCI-AML3^BCOR_KO.24^ were treated for 6 days with increasing doses of 5 different DHODH inhibitors (two-way ANOVA, *n* = 3; *p* < 0.0001****, *p* < 0.001***, *p* < 0.01**, *p* < 0.05*, n.s = non-significant)

We tested two human AML cell lines, OCI-AML2 and OCI-AML3, derived from male patients aged 65 and 57 years, both BCOR wildtype (cellmodelpassports.sanger.ac.uk). Cells were treated with the BCOR inhibitor TP-021 and increasing doses of brequinar (Fig. 1.A). Clonogenic assays showed that 10 µM TP-021 sensitized both lines to brequinar, reducing the SF50 from ~ 25 µM in wildtype cells to ~ 0.1 µM in BCOR-inhibited cells, a 250-fold increase in sensitivity. To confirm BCOR suppression, we examined downstream targets. The Beat AML study [11] reported elevated FOXO1, PDGFA, and JAG1 expression in BCOR-mutant AML. Consistently, TP-021 treatment (6–24 h) upregulated these genes in both OCI-AML2/3 (Figure S.1.D).

We next used a non-malignant hTERT-immortalised RPE1 model and observed a similar phenotype, with a 166-fold increase in brequinar sensitivity upon BCOR inhibition (Fig. 1.A). Collectively, chemical BCOR inhibition upregulates canonical target genes and confers strong brequinar sensitivity, independent of tissue type or malignant state.

To test whether BCOR inhibition broadly sensitizes cells to DHODH blockade, we evaluated four additional inhibitors: leflunomide and teriflunomide, used clinically in multiple sclerosis (MS) and rheumatoid arthritis (RA) [12], and the AML investigational agents AG-636 and farudodstat [13, 14]. OCI-AML2 and OCI-AML3 cells were treated with these compounds ± TP-021 (10 µM) (Figure S.1.C). In all cases, BCOR-inhibited cells showed selective sensitivity, with brequinar and leflunomide most potent at low concentrations (0.01–0.1 µM).

### Synthetic lethality between BCOR and DHODH

To validate synthetic lethality between BCOR dysfunction and DHODH, we first performed siRNA knockdown of BCOR, DHODH, or both in OCI-AML2, OCI-AML3, and RPE1 cells. Single knockdowns had little effect, but combined siBCOR + siDHODH significantly reduced viability across all lines (Fig. 1.B), further enhanced when siBCOR was paired with brequinar. This likely reflects full DHODH inhibition by brequinar versus partial knockdown by siRNA (Figure S.1.E). Live-cell imaging confirmed increased cell death with dual suppression, whether by knockdown or siBCOR plus brequinar (Fig. 1.C–D).

We then tested SKM-1, an AML line harboring a BCOR loss-of-function mutation (c.4977-1G > A; cellmodelpassports.sanger.ac.uk) (Fig. 1.E). Consistent with chemical inhibition and siRNA, genetically BCOR-deficient SKM-1 cells displayed strong sensitivity to all DHODH inhibitors, with brequinar and leflunomide again most effective.

Next, we used CRISPR editing to introduce BCOR loss-of-function mutations in exon 3 of OCI-AML3. Sanger sequencing and Western blot verified edits in three independent clones (BCOR_KO.03, -17 and − 24) (Figure S.1.F–G). All three clones showed synthetic lethality with brequinar, leflunomide, and AG-636 (Fig. 1.F). Attempts to restore BCOR expression caused profound cell death (data not shown), suggesting BCOR-KO cells had adapted to loss of BCOR.

Together, these findings confirm that genetic or pharmacologic DHODH inhibition selectively impairs viability of BCOR-deficient cells, establishing a synthetic lethal relationship.

### Dependency of Bcor-deficient cells on DHODH is due to its role in ros homeostasis

As shown in the supplementary data (Figure S.2), the observed lethality was not attributable to disruptions in the *de novo* pyrimidine synthesis pathway, prompting us to investigate the mitochondrial role of DHODH. By catalyzing the conversion of dihydroorotate to orotate, DHODH reduces ubiquinone (coenzyme Q, CoQ) to ubiquinol, which transfers electrons to complex III of the Electron Transport Chain (ETC). This supports mitochondrial membrane potential and ATP production by sustaining electron flow [15, 16]. Impaired DHODH activity disrupts bioenergetics, elevates reactive oxygen species (ROS), and triggers ferroptosis [10, 18]. In cancer, DHODH is thought to mitigate ROS accumulation, enabling evasion of apoptosis [17]. Moreover, the BCOR^P483L^ mutation in SKM-1 cells has been linked to impaired mitochondrial function and increased ROS [18]. We therefore hypothesized that BCOR-mutant cells exist under ROS stress, and that DHODH depletion may elevate ROS to a lethal threshold.

**Fig. 2:**
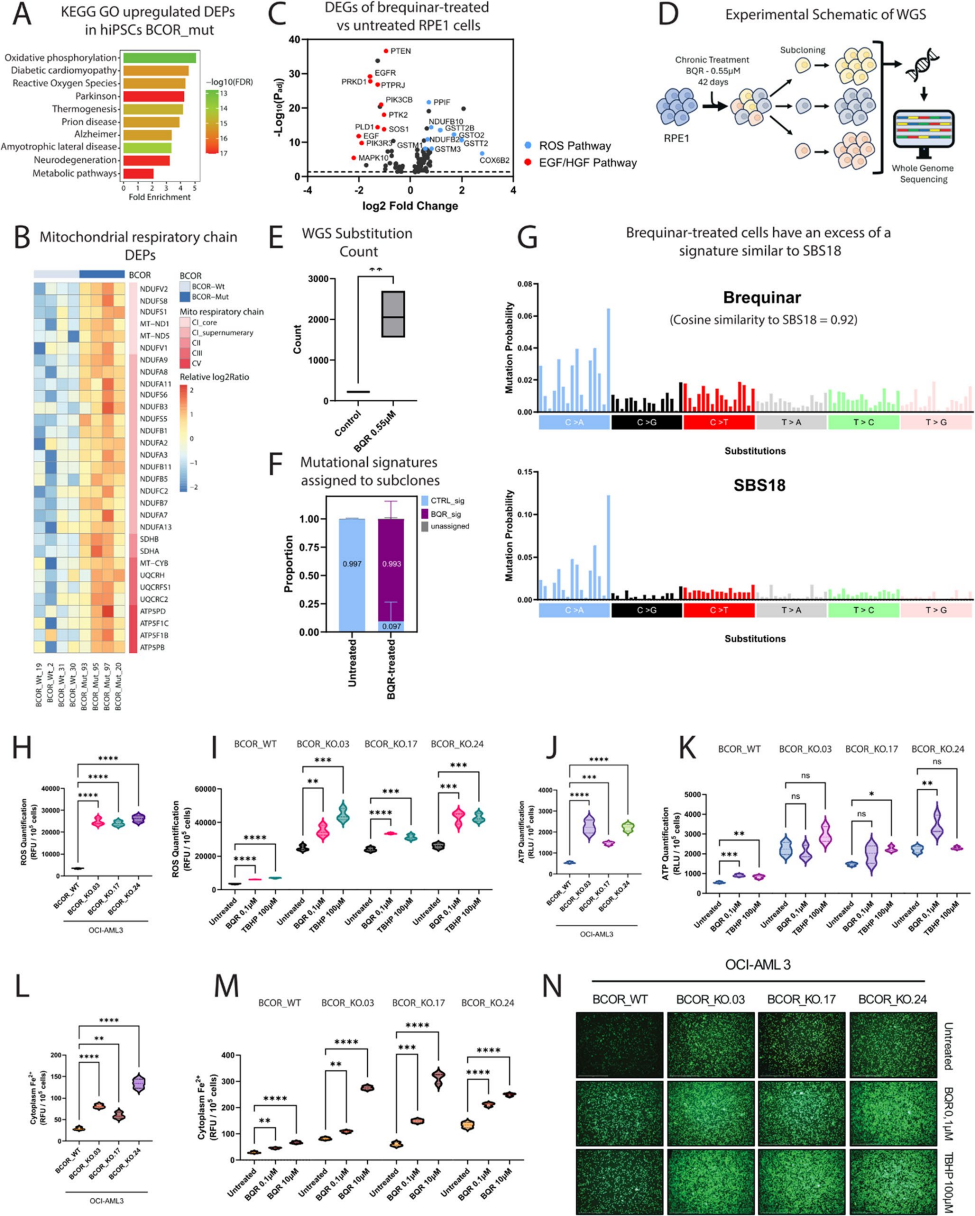
BCOR-mutant cells exhibit higher ROS levels compared to wildtype, with brequinar further elevating this level to potential lethal thresholds. (**A**) Gene Ontology of upregulated Differentially Expressed Proteins (DEPs) in BCOR-mutant contrasted to BCOR-wildtype hiPSCs, showing significant upregulation of Oxidative Phosphorylation, Reactive Oxygen Species and Metabolic Pathways indicating BCOR-mutant cells live in higher oxidative stress environment. (**B**) Heatmap of DEPs involved in mitochondrial electron transport chain (ETC) in BCOR-mutant and Wildtype hiPSCs. This significant increase of ETC-involved gene expression in BCOR-mutant cells indicate higher mitochondrial metabolism and potentially higher oxidative stress. (**C**) Volcano plot of significantly differentially expressed genes (DEGs) (-Log_10_[P_adj_] > 1,30) RPE1 cells treated with brequinar (0.55µM, 24 h) versus untreated, highlighting upregulation of ROS-pathway genes and downregulation of EGF/HGF-pathway genes. (**D**) Experimental schematic of Whole Genome Sequencing experiment. RPE1 cells were chronically treated with brequinar 0.55µM or untreated for 42 days, then subcloned and amplified for WGS. (**E**) Single base substitution counts comparing 3 untreated RPE1 clones to 3 brequinar-treated RPE1 clones. (SBS count plot, t-test, *n* = 3; *p* < 0.01**). (**F**) Exposure proportions of brequinar and control signatures, showing specific enrichment of brequinar signature in brequinar-treated RPE1 cells. (**G**) Single base substitution plots showing a high similarity of brequinar signature to SBS18 (Cosine similarity = 0.92) (Degasperi et al., 2022), which is induced by an excess of 8-oxo-dG indicating oxidative damages in DNA. (**H**) Basal ROS levels amongst OCI-AML3^BCOR_WT^, OCI-AML3^BCOR_KO.03^, OCI-AML3^BCOR_KO.17^ and OCI-AML3^BCOR_KO.24^. BCOR-mutant cells show significantly higher basal ROS level compared to wildtype (Relative fluorescent units per 100,000 cells - two-way ANOVA, *n* = 3; *p* < 0.0001****). (**I**) DHODH-inhibition through brequinar treatment further exacerbate ROS levels in OCI-AML3^BCOR_WT^, OCI-AML3^BCOR_KO.03^, OCI-AML3^BCOR_KO.17^ and OCI-AML3^BCOR_KO.24^ (two-way ANOVA, *n* = 3; *p* < 0.0001****, *p* < 0.001***, *p* < 0.01**). (**J**) Basal ATP levels amongst OCI-AML3^BCOR_WT^, OCI-AML3^BCOR_KO.03^, OCI-AML3^BCOR_KO.17^ and OCI-AML3^BCOR_KO.24^. BCOR-mutant cells show significantly higher basal ATP level compared to wildtype, possibly a sign of enhanced oxidative phosphorylation (Relative luminescent units per 100,000 cells - two-way ANOVA, *n* = 3; *p* < 0.0001****, *p* < 0.001***). (**K**) DHODH-inhibition through brequinar treatment further exacerbate ATP levels in OCI-AML3^BCOR_WT^, but not BCOR-mutant cell lines, implying that BCOR-mutant cells may be operating at their saturating limit of metabolic ATP production (two-way ANOVA, *n* = 3; *p* < 0.001***, *p* < 0.01**, *p* < 0.05*, n.s = non-significant). (**L**) Basal cytoplasmic Fe^2+^ levels amongst OCI-AML3^BCOR_WT^, OCI-AML3^BCOR_KO.03^, OCI-AML3^BCOR_KO.17^ and OCI-AML3^BCOR_KO.24^. BCOR-mutant cells show significantly higher basal cytoplasmic Fe^2+^ level compared to wildtype (Relative fluorescent units per 100,000 cells - two-way ANOVA, *n* = 3; *p* < 0.0001****). (**M**) DHODH-inhibition through brequinar treatment further exacerbate cytoplasmic Fe^2+^ levels in OCI-AML3^BCOR_WT^, OCI-AML3^BCOR_KO.03^, OCI-AML3^BCOR_KO.17^ and OCI-AML3^BCOR_KO.24^, where accumulation of Fe^2+^ is directly linked to Ferroptosis (two-way ANOVA, *n* = 3; *p* < 0.0001****, *p* < 0.001***, *p* < 0.01**). (**N**) Representative images of cells captured under a fluorescence microscope using GFP excitation at 10× magnification, highlighting reactive oxygen species (ROS) as GFP fluorescence. Scale bar = 500 μm

Supporting this, Gene Ontology analysis of published BCOR-mutant hiPSC proteomic and transcriptomic data [8] revealed upregulation of oxidative phosphorylation and ROS production genes (Fig. 2.A – S.3.A), with increased expression of components of all five ETC complexes (Fig. 2.B – S.3.B). Metallothioneins, ROS scavengers induced under oxidative stress [19], were also elevated (Figure S.3.C). These findings suggest BCOR-mutant iPSCs experience basal oxidative stress.

We next examined whether DHODH inhibition elevates ROS. RPE1 cells treated with brequinar at its EC50 (0.55 µM) for 24 h showed increased expression of ROS-generating genes and decreased expression of Epidermal Growth Factor (EGF) and Hepatocyte Growth Factor (HGF) pathway genes (Fig. 2.C – S.3.E), consistent with impaired proliferation under oxidative stress [20]. Upregulated transcripts included PPIF and NDUFB2–10 (ROS production), as well as ROS scavengers COX6B2, GSTO2, GSTT2/2B, GSTM1, and GSTM3 (Fig. 2.C – S.3.F–G).

Chronic brequinar treatment (42 days) of RPE1 cells induced a ten-fold increase in Single Base Substitution count (Fig. 2.E) with mutational signature SBS18, linked to oxidative lesions and 8-oxo-dG accumulation [21] (Fig. 2.F–G). Together, these findings show that DHODH inhibition elevates ROS independently of BCOR.

We next evaluated whether synthetic lethality arises from DHODH inhibition exacerbating ROS in oxidatively stressed BCOR-mutant cells.

### DHODH inhibition induces potentially lethal oxidative stress in BCOR-mutant cells

We compared ROS levels in OCI-AML3 BCOR-wildtype and three BCOR-mutant clones following 24 h brequinar treatment. TBHP, a glutathione inhibitor and ROS inducer, served as a positive control. At baseline, BCOR-mutant clones showed significantly higher ROS than wildtype (Fig. 2.H). Even low-dose brequinar (0.1 µM – 24 h) markedly increased ROS, approaching TBHP-induced levels (3 h of treatment) (Fig. 2.I), indicating DHODH inhibition exacerbates ROS stress in BCOR-mutant OCI-AML3.

Notably, TBHP treatment for six days did not induce cell death, unlike DHODH inhibition, suggesting DHODH’s role extends beyond ROS scavenging to sustaining mitochondrial metabolism (Figure S.3.J). Supporting this, BCOR-mutant clones displayed elevated baseline ATP compared to wildtype (Fig. 2.J). Brequinar increased ATP in wildtype but not mutant clones (Fig. 2.K), consistent with BCOR-deficient cells already operating at maximal ATP output and crossing a survival threshold under DHODH inhibition.

Because ferroptosis has been linked to DHODH inhibition [17], we examined Fe²⁺ accumulation. BCOR-mutant cells showed higher baseline Fe²⁺ than wildtype (Fig. 2.L). Brequinar (0.1 or 10 µM, 24 h) further elevated Fe²⁺ in both groups, supporting ferroptosis induction. Transcriptomics from brequinar-treated RPE1 cells confirmed increased expression of pro-ferroptosis genes (Figure S.3.H) and decreased expression of protective genes (Figure S.3.I).

## Discussion

**Fig. 3:**
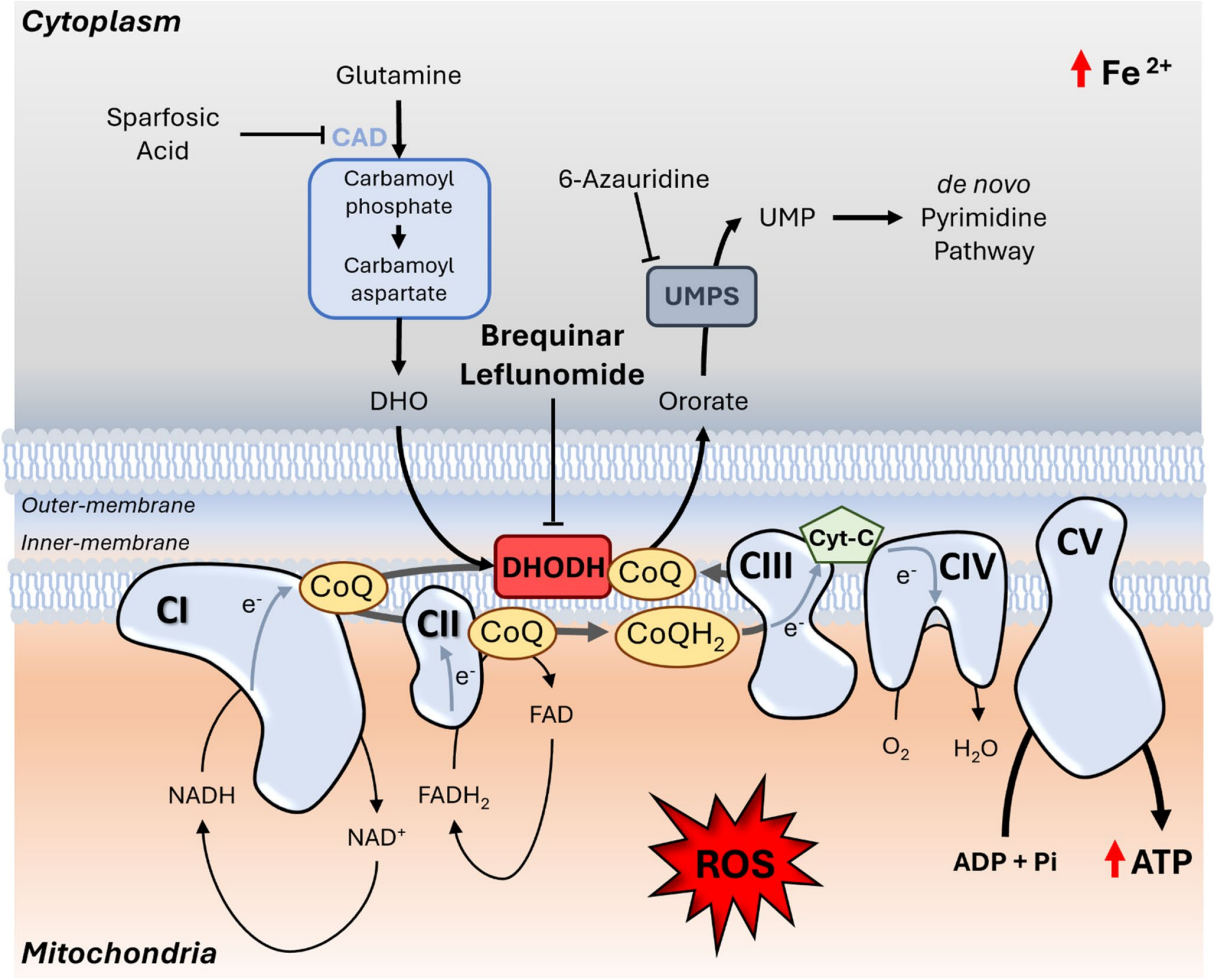
Graphical summary of DHODH-inhibitors in BCOR-mutant cells. Here, we demonstrate that BCOR-deficient cells exhibit heightened sensitivity to DHODH inhibitors, such as brequinar. DHODH is a key enzyme in the de novo pyrimidine synthesis pathway, responsible for converting dihydroorotate to orotate, ultimately leading to the production of dUTP, dCTP, and dTTP necessary for DNA replication. When BCOR-deficient cells were treated with inhibitors targeting other components of the pathway, such as CAD-inhibitor (sparfosic acid) or UMPS-inhibitor (6-azauridine), the same sensitivity seen with DHODH inhibitors was not replicated. This led us to hypothesize that the DHODH dependency in BCOR-deficient cells is linked to its role within the mitochondrial Electron Transport Chain (ETC). DHODH reduces ubiquinone (CoQ) to ubiquinol (CoQH2), which subsequently facilitates electron transfer from complexes I and II to complex III. BCOR-mutant cells show increased ROS levels compared to wild-type cells, which are further amplified by brequinar treatment and associated with enhanced ATP production and elevated Fe^2^ + levels. Thus, brequinar and other DHODH inhibitors are effective tools for selectively increasing ROS in BCOR-deficient cells, thereby inducing targeted cell death. CAD (carbamoyl-phosphate synthetase 2, aspartate transcarbamylase, dihydroorotase); DHO (dihydroorotate); DHODH (dihydroorotate dehydrogenase); UMPS (uridine monophospate synthetase); UMP (uridine monophosphate); CoQ (coenzyme Q - ubiquinone); CoQH_2_ (ubiquinol); Cyt-c (cytochrome c); ROS (reactive oxygen species) (adapted from Boukalova et al. [9]).

In this study, we identify a synthetic lethal interaction between *BCOR* and *DHODH*, uncovering a therapeutic vulnerability in *BCOR*-mutant cells across multiple cancer and cell models. We establish a class effect for DHODH inhibitors, confirmed by both chemical inhibition and genetic manipulation, with efficacy varying among compounds. Mechanistically, lethality stems from *DHODH*’s role in controlling ROS (Fig. 3).

Altogether, our results position BCOR mutations as a potential genomic biomarker that may help pave the way toward repurposing DHODH inhibitors and advancing precision medicine strategies in AML and other cancers; and we hope this preliminary study will stimulate deeper investigations into high-ROS cancers and the mechanisms underlying DHODH vulnerability.

Limitations of our study include the need for further mechanistic investigations and the absence of in vivo and primary patient sample models to fully validate our findings. However, DHODH inhibitors already have strong clinical and preclinical support, including AML trials where they show activity in resistant patients. A screen of > 330,000 compounds in a murine AML model also identified DHODH inhibitors as predominant hits [10]. Our work introduces *BCOR* loss as a biomarker for DHODH therapy, relevant since *BCOR* mutations are poor prognostic markers in AML and other cancers [22].

Several DHODH inhibitors are clinically advanced: AG-636 shows antitumor activity in lymphoma models (10–100 mg/kg BID, 14 days) and has entered Phase 1 testing (NCT03834584) [13]. Leflunomide, approved for autoimmune disease, is dosed at 20 mg/day (~ 74 µM). Brequinar is orally bioavailable and under evaluation in AML (NCT03760666) and COVID-19 (NCT04425252) at ~ 0.3 mM daily. Notably, these doses are much higher than those effective in our *BCOR*-mutant cell models.

We further show that *BCOR*-deficient cells exhibit elevated ROS and ATP compared to wildtype. DHODH inhibition increases ROS, but in *BCOR*-mutant cells this fails to trigger compensatory ATP production, suggesting they already operate at their metabolic ceiling. This metabolic stress likely underlies the synthetic lethality observed in BCOR-mutant cells, potentially in combination with pyrimidine disruption induced by DHODH inhibition.

Clinically, *BCOR* mutations may also impact responses to other therapies: they cooperate with *FLT3* mutations to drive FLT3 inhibitor resistance [23]. Supporting this, brequinar sensitized FLT3-mutant MOLM13 cells (Supplementary Figure S.4.A). *BCOR* mutations are also enriched in salivary gland and endometrial cancers (Supplementary Figure S.4.B), extending DHODH inhibitors’ potential beyond AML. Preclinical studies combining brequinar with venetoclax in high-grade B-cell lymphoma further highlight therapeutic promise [24].

## Supplementary Information

Below is the link to the electronic supplementary material.

Supplementary Material 1 (DOCX 26.9 KB)

Supplementary Material 2 (DOCX 1.66 MB)

## Data Availability

No datasets were generated or analysed during the current study.
