# Supplementary material for: BCOR mutations define a therapeutic vulnerability to DHODH Inhibition in acute myeloid leukemia
Source: Ann Hematol. 2026 Jan 19;105(1):32. doi: 10.1007/s00277-026-06773-z (PMC12812770; doi:10.1007/s00277-026-06773-z)
Supplement: Supplementary file 1 — Supplementary Material 1 (DOCX 26.9 KB) [file 277_2026_6773_MOESM1_ESM.docx]

**BCOR Mutations Define a Therapeutic Vulnerability to DHODH Inhibition in Acute Myeloid Leukemia**

**AUTHORS**

Florian Robert^1,2^, Cherif Badja^1,2^, Soraya Boushaki^1,2^, Andrea Degasperi^1,2^, Yasin Memari^1,2^, Sophie Momen^1,2^, Theodoros I. Roumeliotis^4^, Zuza Kozik^4^, Malgorzata Gozdecka ^3^, Jyoti Choudhary^4^, George Vassiliou^3^, Gene CC Koh^1,2^, Serena Nik-Zainal^1,2^

**METHODS**

**CELLULAR MODELS MAINTENANCE:**

**Suspension cells:**

All cells were cultured in T75 flasks. OCI-AML2 and -3 were cultured in alpha-MEM with 20% FCS and 1% PSG. MOLM13 were cultured in in RPMI FCS 10% PSG 1%, HL60 cells in RPMI + FCS 20% + Glutamine 2 mM, and SKM-1 cells in RPMI + FCS 15%. Passages were performed every 2-3 days at 1/5 dilution. All components were provided by Gibco™

**RPE1 cells:**

RPE1 cells were cultured in T75 flasks in DMEM F12 (Gibco™) with 10% FCS. Every 3 days, medium was changed. Once at confluency, cells were trypsinized and amplified in new T75 flasks at 1/5 dilution.

**hiPSCs:**

hiPSCs were maintained under feeder-free conditions on Vitronectin FX^TM^ (STEMCELL Technologies) coated plates cultured and fed with Essential E8^TM^ medium (Gibco™). Fresh medium replacement was performed daily, and the cell lines were checked to ensure they were healthy, with no visible signs of spontaneous differentiation. For cell propagation, hiPSCs were treated with 0.5mM UltraPure^TM^ EDTA (Invitrogen^TM^), rinsed with DPBS (Gibco™) and passaged as small cell clumps.

**siRNA TRANSFECTION AND TP-021 TREATMENT, RNA EXTRACTION, RT-QPCR**

**TP-021 treatment:**

OCI-AML 2 and -3 cells were seeded in 6-well plates at 1 million cells per well in alpha-MEM with 20% FCS and 1% PSG, then treated for 6 or 24 hours. After treatment, cells were harvested, centrifuged (5 min, 150g, 4°C), washed with PBS, centrifuged again (10 min, 10,000g, 4°C), and frozen at -80°C for subsequent RNA extraction.

RPE1 cells were seeded in 6-well plates at 600,000 cells per well in DMEM F12 with 10% FCS and allowed to settle overnight. They were then treated for 6 or 24 hours and processed as described for OCI-AML cells.

**siRNA transfection:**

OCI-AML 2 and -3 cells were seeded in 6-well plates at 30-50% confluence in alpha-MEM with 20% FCS, without PSG. siRNA was prepared according to the Thermo Fisher Lipofectamine 2000 protocol (<https://www.thermofisher.com/uk/en/home/references/protocols/cell-culture/transfection-protocol/stealth-sirna-transfection-lipofectamine.html>). Cells were allowed to incubate for 72 hours, then harvested, centrifuged (5 min, 150g, 4°C), washed with PBS, centrifuged again (10 min, 10,000g, 4°C), and frozen at -80°C for further RNA extraction. RPE1 cells were seeded similarly and processed in the same way as the OCI-AML cells.

**siRNA:**

siRNAs were purchased from ThermoFisher website.

- BCOR: ID 132062 Catalog AM16708
- DHODH: ID 112770 Catalog AM16708
- Silencer™ Negative Control No. 1 siRNA AM4611

**RNA Extraction:**

Total RNA was extracted from cells using the Direct-Zol™ RNA Miniprep Plus Kit (Zymo Research) according to the manufacturer’s instructions. Briefly, cell pellets were resuspended in 100 µL PBS and lysed directly in TRI Reagent (Invitrogen). The lysate was passed through a Zymo-Spin column to bind RNA. DNase I (Zymo Research) was used to degrade any DNA, and the columns were washed to remove contaminants. The RNA was then eluted in RNase-free water. RNA quality and concentration were assessed using a NanoDrop spectrophotometer.

cDNA synthesis was performed using 1.5 µg of total RNA and the SuperScript IV VILO Master Mix (Thermo Fisher Scientific) in a 200 µL reaction, following the manufacturer's protocol. The RNA was combined with the VILO master mix and incubated at 25°C for 10 minutes, 50°C for 10 minutes, and 85°C for 5 minutes to inactivate the reverse transcriptase.

**RT-qPCR:**

Quantitative PCR was conducted in a 96-well plate format to assess the expression of target genes in cDNA samples. Each reaction included TaqMan Fast Advanced Master Mix (ThermoFisher Scientific), 1 µL of cDNA, and gene-specific TaqMan assays (ThermoFisher Scientific) as listed below. Each reaction was performed in 3 to 6 technical replicates to ensure reproducibility. The plates were analyzed using a real-time PCR system (QuantStudio3 - ThermoFisher), with the following cycling conditions:

- Activation 50°C for 2 minutes

- Initial denaturation at 95°C for 20 seconds

- 40 cycles of 95°C for 1 second and 60°C for 20 seconds

Data were analysed using the ΔΔCt method to determine relative gene expression levels normalized to a housekeeping gene (GAPDH). The results were plotted as fold changes relative to control conditions.

Taqman probes were purchased from ThermoFisher website:

- BCOR: ID Hs00372378_m1 #4331182
- DHODH: ID Hs00361406_m1 #4331182
- FOXO1: ID Hs00231106_m1 #4331182
- PDGFA: ID Hs00234994_m1 #4331182
- JAG1: ID Hs01070032_m1 #4331182
- GAPDH: ID Hs02786624_g1 #4331182
- CAD: ID Hs00983188_m1 #4331182
- UMPS: Hs00923517_m1 #4331182

TAQMAN FATS ADVANCED MMIX 5ML - #4444557

**siRNA TRANSFECTION AND SURVIVAL QUANTIFICATION**

OCI-AML 2 and -3 cells were seeded in 96-well plates at 30-50% confluence in alpha-MEM + FCS 20% without PSG. siRNAs were prepared as described earlier. After treatment, the cells were lysed, and cell viability was measured according to Promega’s CellTiter-Glo protocol.

RPE1 cells were seeded in 96-well plates at 30-50% confluence in DMEM F12 + FCS 10% and allowed to settle overnight. The cells were then processed in the same manner as OCI-AML cells.

**CELL TREATMENT AND SURVIVAL QUANTIFICATION**

OCI-AML 2 and -3 cells were seeded in 96-well plates at 20% confluence in alpha-MEM + FCS 20% + PGS 1%. MOLM13 cells were seeded similarly in RPMI FCS 10% PSG 1%, HL60 cells in RPMI + FCS 20% + Glutamine 2 mM, and SKM-1 cells in RPMI + FCS 15%. Treatment was applied during seeding. Cells were allowed to grow for 6 days, and the readout was processed following Promega’s CellTiter-Glo protocol.

RPE1 cells were seeded in 96-well plates at 20% confluence in DMEM F12 + FCS 10% and allowed to settle overnight. Treatment was applied during seeding, and the cells were then processed in the same manner as OCI-AML cells.

**INCUCYTE CELL PROLIFERATION**

RPE1 cells were seeded in 12-well plates at 40% confluency in DMEM F12 + FCS 10% and allowed to settle overnight. SiRNAs were then prepared as described earlier. The Incucyte system was set to Phase contrast at 10x magnification, capturing 2 images per well every 12 hours. Analysis was performed using the Basic Analyzer protocol.

**REAGENTS AND CELL LINES**

Following compounds were purchased from Cayman Chemical:

- Brequinar ID 24445

Following compounds were purchased from MedChemExpress, as 10mM in DMSO:

- TP-021 - Cat. No.: HY-119402
- Leflunomide - Cat. No.: HY-B0083
- Teriflunomide - Cat. No.: HY-15405
- AG-636 - Cat. No.: HY-137463
- Farudodstat - Cat. No.: HY-129239
- Sparfosic Acid - Cat. No.: HY-112732B

6-Azauridine was purchased on ThermoFisher website – CAS 54-25-1

All suspension cell lines OCI-AML 2, -3, MOLM13 and HL60 were kindly gifted from Pr. George Vassiliou. SKM-1 were purchased from DSMZ (ACC 547).

**STATISTICAL ANALYSIS**

Statistical analysis was performed using t-test, one-way ANOVA, and two-way ANOVA, depending on the experimental design. A t-test was used to compare the means between two groups for experiments with a single independent variable. One-way ANOVA was applied to analyze differences among multiple groups under a single treatment condition. For experiments involving two independent variables, two-way ANOVA was conducted to evaluate interactions between the variables and their effect on the dependent variable. Post-hoc tests, such as Tukey's test, were used to assess pairwise differences when ANOVA results were significant. Statistical calculations and graphs were done using GraphPad Prism 10 (GraphPad Software Inc). In all analyses, a 2-tailed p-value <0.05 was considered statistically significant: p<0.05*; p<0.01**; p<0.001*** and p<0.0001****.

**CRISPR – CAS9**

Solutions were prepared prior to nucleofection. All compounds were purchased from:

- Integrated DNA Technologies:
  - Alt-R™ S.p. HiFi Cas9 Nuclease V3, 100 µg - 1081058
  - Alt-R™ Cas9 Elec Enhancer, 100 nmol – 10007805
  - Templates
    - BCOR_KO: insertion of frameshift mutation - Sequence: /Alt-R-HDR1/C*T* CTT CCC TCA GTT CCA GTC TGG CTT TTG AAG CGT CAC CAT CAT TTG CTC TGT TGG ACA AGG ATT TTC CTA TTT AAA AAG ATA CAC CAA CTT CA*T* G/Alt-R-HDR2/
    - BCOR_rescue: Sequence: /Alt-R-HDR1/G*A* TTC TCT TCC CTC AGT TCC AGT CTG GCT TTT GAA GCG TCA CCA TCA TTT ACA AGG ATT TTC CTA TTT AAA AAG ATA CAC CAA CTT CAT GAA* A*G/Alt-R-HDR2/
- Lonza:
  - SG Cell Line 4D-Nucleofector™ X Kit L - Catalog #: V4XC-3024
- Synthego:
  - sgRNA BCOR_KO: aagcgtcaccatcatttaca
  - sgRNA BCOR_rescue: gtcaccatcatttgctctgt
- SelleckChem:
  - Nedisertib (M3814) - Catalog No.S8586
- Gibco:
  - Opti-MEM™ I Reduced Serum Medium – 31985062
- Additionnal material:
  - Lonza 4D-Nucleofector® X and Core Unit

The Cas9 complex solution was prepared in advance by diluting in freshly prepared PBS with sgRNA. The mixture was incubated at room temperature for 30 minutes to allow complex formation, after which templates were added. OCI-AML3_Cas9 cells were spun down at 150g for 5 minutes. The medium was discarded, and cells were resuspended in 1 mL of DPBS. After counting, 200,000 cells were used for each electroporation. The required cell amount was washed in 10 mL of DPBS and spun down at 100g for 10 minutes. The supernatant was discarded, and the cell pellet resuspended in 20 µL of SG solution, supplemented with enhancer. Five µL of Cas9 solution was added to this mix. The volume was transferred to a 16-well Lonza strip for electroporation, using the EH-100 program. The strip was left at room temperature for 5 minutes. Afterward, 80 µL of warm OptiMEM was added to each well, and the strip was incubated at 37°C for 10 minutes. Finally, 50 µL from each well was then seeded into a 12-well plate (50 µL per well, approximately 100,000 cells) containing warm culture medium (alpha-MEM FCS 20% PSG 1%) and supplemented with 0.4 µL of M3814 per 1 mL of medium for BCOR_KO.

**dNTPS AND UMP QUANTIFICATION**

**dNTP and UMP extraction**

OCI-AML cells were seeded at 10^6 in 10 mL of alpha-MEM FCS 20% PSG 1%. After 24 hours of treatment, the cells were spun down for 5 minutes at 150g. The medium was discarded, and cells were resuspended in 1 mL of DPBS for counting, which was used to normalize dNTP and UMP concentrations. Next, 9 mL of ice-cold DPBS was added to the pellet, and the cells were centrifuged at 4°C for 5 minutes at 3000g. The supernatant was discarded, and the pellet was resuspended in 500 µL of ice-cold 60% methanol, then transferred to an Eppendorf tube. Cells were vortexed and heated at 95°C for 3 minutes, followed by centrifugation at 16,000g for 5 minutes at 4°C. Meanwhile, Amicon Ultra 0.5 mL filters were equilibrated with ice-cold 60% methanol. After centrifugation, the supernatant was collected and filtered through Amicon Ultra 0.5 mL filters to collect dNTPs or UMP. This was centrifuged again at 16,000g for 5 minutes at 4°C and repeated as necessary. The thin pellet was left to dry and resuspended in 100 µL of nuclease-free water. dNTPs were quantified using a Nanodrop on the ssDNA mode. The samples were stored at -80°C for future use.

**dNTPs Quantification**

Quantification was performed according to the protocol published by Purhonen *et al.* 2020 (10.1093/nar/gkaa516) and detailed protocol can be found as supplementary data (<https://academic.oup.com/nar/article/48/15/e87/5861405>).

**UMP Quantification**

Quantification has been performed accordingly to protocol established by Promega in UMP/CMP-Glo™ Glycosyltransferase Assay (CAT# VA1130)

**RNA SEQUENCING AND ANALYSIS**

**RNA sequencing:**

Total RNA was isolated using the PureLink RNA Mini Kit (ThermoFisher) following the manufacturer’s instructions. cDNA libraries were then constructed and sequenced on the Illumina Novaseq 6000 platform, generating over 20 million 150 bp paired-end read pairs per sample.

**RNAseq analysis:**

Splice-aware aligner STAR v2.5.0a was used to map the reads to the reference genome. A genome index was built for the human decoy reference genome hs37d5.fa.gz, and fastq files were mapped using splice junction data from the Gencode GTF annotation file v19. Read fragments associated with gene features were quantified using featureCounts v2.0.1. Raw count matrices were subsequently analyzed in R v4.0.4, and differential gene expression was determined using the DESeq2 R package. KEGG GO analyses were performed using ShinyGO 0.82.

**WHOLE GENOME SEQUENCING FOR MUTATIONAL SIGNATURE ANALYSIS**

Treated and untreated RPE1 cells were single-cell subcloned prior to whole genome sequencing. A total of 50 cells resuspended in 10 mL DMEM FCS 10% were distributed into a 96-well plate (100 µL per well) and incubated at 37°C with 5% CO2. After 24 hours, the plate was inspected under a microscope to identify wells with single-cell-derived clones. Once the clones reached 70-100% confluence, they were expanded for approximately one month (starting from single-cell subcloning) before being collected for whole genome sequencing. Three subclones from both Brequinar-treated and untreated conditions were selected for further processing.

Genomic DNA from human RPE1 cells was extracted using the GenElute™ Mammalian Genomic DNA Miniprep Kits (Sigma-Aldrich, GIN350) according to the manufacturer’s instructions. DNA samples passing quality control were sequenced using the Illumina X-10 platform (version 4), generating 120G of raw data with 150 paired-end nucleotides. The sequencing coverage was 30x for whole genome sequencing. Reads from each sample were mapped to the reference genome of *Homo sapiens* (hg38), and mutations were called by comparing the subclones (treated and untreated) to their parental matched RPE1 cells.

For mutational signature analysis, single base substitutions were called using the Cancer Variants Through Expectation Maximization (CaVEMan) algorithm. Only mutations private to each of the subclones were used. The 96-channel single substitution catalogues were generated using the "Analyse" tool on the mutational signature analysis website (<https://signal.mutationalsignatures.com/analyse2>), and the profiles were visualized using R software. A control mutational signature was obtained by averaging the catalogue obtained from the untreated clones. The Brequinar mutational signature was obtained by subtracting the mean catalogue obtained from the untreated clones from the mean catalogue obtained from the treated samples. Exposures of control and Brequinar signatures were then estimated in each untreated and treated clones using the Fit algorithm from the signature.tools.lib R package (<https://github.com/Nik-Zainal-Group/signature.tools.lib>) with default parameters.

**ROS, ATP and Fe^2+^ QUANTIFICATION**

**ROS Quantification:**

OCI-AML3^BCOR_WT^ and OCI-AML3^BCOR_KO^ were seeded at 100,000 cells per well in 24-well plates. On day 1, Brequinar treatment was applied for 24 hours. On day 2, TBHP (provided in the kit) treatment was administered for 3 hours. Prior to the protocol provided by OZ-Biosciences (ROS0300), cells were counted for each well. We then followed the standard protocol for suspension cells to quantify ROS using a Pherastar plate reader. Prior to quantification, pictures were taken with Invitrogen EVOS FL Auto 2 Imaging System, 10x magnification

**ATP Quantification:**

OCI-AML3^BCOR_WT^ and OCI-AML3^BCOR_KO^ were seeded at 10,000 cells per well in 96-well black plates. On day 1, Brequinar treatment was applied for 24 hours, and on day 2, TBHP treatment was applied for 3 hours. Cells were counted for each well before proceeding with the protocol outlined by Promega’s CellTiter-Glo. We followed the manufacturer's instructions and quantified Relative Luminescent Units using a Pherastar plate reader.

**Cyoplasm Fe^2+^ Quantification:**

OCI-AML3^BCOR_WT^ and OCI-AML3^BCOR_KO^ were seeded at 100,000 cells per well in 24-well plates. On day 1, Brequinar treatment was applied for 24 hours. Prior to the protocol provided by Merck for FerroOrange labelling (SCT210-35nmol – Protocol available as Data Sheet), cells were counted for each well. We then followed the standard protocol to quantify Fe^2+^ using a Pherastar plate reader. Prior to quantification, pictures were taken with Invitrogen EVOS FL Auto 2 Imaging System, 10x magnification

**PROTEOMICS**

Cell pellets were lysed in a buffer containing 1% sodium deoxycholate (SDC), 100 mM triethylammonium bicarbonate (TEAB), 10% isopropanol and 50 mM NaCl, freshly supplemented with 5 mM TCEP (Thermo, Bond-breaker), 10 mM iodoacetamide (IAA), universal nuclease 1:2000 v/v (Pierce, #88700) and Halt protease and phosphatase inhibitor cocktail (Thermo, #78442, 100X) with 5 min of bath sonication. Protein concentration was measured with the Quick Start Bradford protein assay (Bio-Rad). Aliquots of 30 μg of total protein were digested overnight with trypsin (Pierce, 1:20) at room temperature. Peptides were labelled with the TMT-11plex reagents (Thermo) by adding 10 μL of the reagent (20 μg/μL) into 25 μL of sample volume. The TMT mixture was acidified with formic acid at 2% and the precipitated SDC was removed by centrifugation. “Pooled control” samples were made by combining 50 μg of total protein from each of the control samples, divided into six aliquots of 30 μg each and processed as above, to be used as bridge samples between TMT batches.

Peptides were fractionated offline with high-pH Reversed-Phase (RP) chromatography on the XBridge C18 column (2.1 x 150 mm, 3.5 μm, Waters) on a Dionex UltiMate U3000 HPLC system. 0.1% (v/v) ammonium hydroxide was used as mobile phase A and 0.1% (v/v) ammonium hydroxide in acetonitrile was used as mobile phase B. The TMT labelled peptides were fractionated with a gradient elution method at 0.2 mL/min, incorporating the following steps: for 5 minutes isocratic at 5% B, for 35 min gradient to 35% B, for 5 min gradient to 80% B, isocratic for 5 minutes and re-equilibration to 5% B. Fractions were collected every 42 sec, pooled into 30 samples, and SpeedVac dried. LC-MS analysis was performed on a Dionex UltiMate U3000 UHPLC system coupled with the Orbitrap Lumos Mass Spectrometer (Thermo Scientific). 40 μL 0.1% formic acid was used to reconstitute each peptide fraction and 10 μL was loaded to the Acclaim PepMap 100, 100 μm × 2 cm C18, 5 μm, trapping column at a flow rate of 10 μL/min. Samples were analysed with the EASY-Spray C18 capillary column (75 μm × 50 cm, 2 μm) at 50 °C. Mobile phase A was 0.1% formic acid and mobile phase B was 80% acetonitrile, 0.1% formic acid. The elution method was: for 95 min gradient 5%-38% B, for 5 min up to 95% B, for 5 min isocratic at 95% B, re-equilibration to 5% B in 5 min, for 10 min isocratic at 5% B at a flow rate of 300 nL/min. Precursor ions were selected in the range of 375-1,500 m/z with mass resolution of 120 k, AGC 4×10^5 and max IT 50 ms with the top speed mode in 3 sec and were isolated for CID fragmentation with quadrupole isolation width 0.7 Th. Collision energy was 35% with AGC 2×10^4 and max IT 50 ms. Quantification was obtained at the MS3 level with HCD fragmentation of the top 5 most abundant CID fragments isolated with Synchronous Precursor Selection (SPS). Quadrupole isolation width was 0.7 Th, collision energy was 55% and AGC setting 1×10^5 with 100 ms max IT. The HCD MS3 spectra were acquired for the mass range 100-500 m/z with 50k resolution. Targeted precursors were dynamically excluded for 45 seconds with 7 ppm mass tolerance.

The SequestHT search engine in Proteome Discoverer 2.4 (Thermo Scientific) was used to analyse the mass spectra for protein identification and quantification. The precursor and fragment ion mass tolerances were 20 ppm and 0.5 Da respectively. Spectra were searched for fully tryptic peptides with maximum 2 missed cleavages. Static modifications were TMT6plex at N- terminus/K and Carbamidomethyl at C. Dynamic modifications were Oxidation of M and Deamidation of N/Q. Peptide confidence was estimated with the percolator node and peptides were filtered at q-value<0.01 based on target-decoy database search. All spectra were searched against reviewed UniProt human protein entries. The reporter ion quantifier node included a TMT-11plex quantification method with an integration window tolerance of 15 ppm at the MS3 level. Only unique peptides were used for quantification, considering protein groups for peptide uniqueness. Only peptides with average reporter signal-to-noise>3 were used for protein quantification. Raw protein abundances were normalized to sample median, log2 transformed, scaled to the average of the reference pooled samples per TMT batch, and centered at zero across all samples. Differential analysis was performed with the Bioconductor HybridMTest package in RStudio.
