# Supplementary material for: BCOR mutations define a therapeutic vulnerability to DHODH Inhibition in acute myeloid leukemia
Source: Ann Hematol. 2026 Jan 19;105(1):32. doi: 10.1007/s00277-026-06773-z (PMC12812770; doi:10.1007/s00277-026-06773-z)
Supplement: Supplementary file 2 — Supplementary Material 2 (DOCX 1.66 MB) [file 277_2026_6773_MOESM2_ESM.docx]

**BCOR Mutations Define a Therapeutic Vulnerability to DHODH Inhibition in Acute Myeloid Leukemia**

**AUTHORS**

Florian Robert^1,2^, Cherif Badja^1,2^, Soraya Boushaki^1,2^, Andrea Degasperi^1,2^, Yasin Memari^1,2^, Sophie Momen^1,2^, Theodoros I. Roumeliotis^4^, Zuza Kozik^4^, Malgorzata Gozdecka ^3^, Jyoti Choudhary^4^, George Vassiliou^3^, Gene CC Koh^1,2^, Serena Nik-Zainal^1,2^

**SUPPLEMENTARY FIGURES**

^
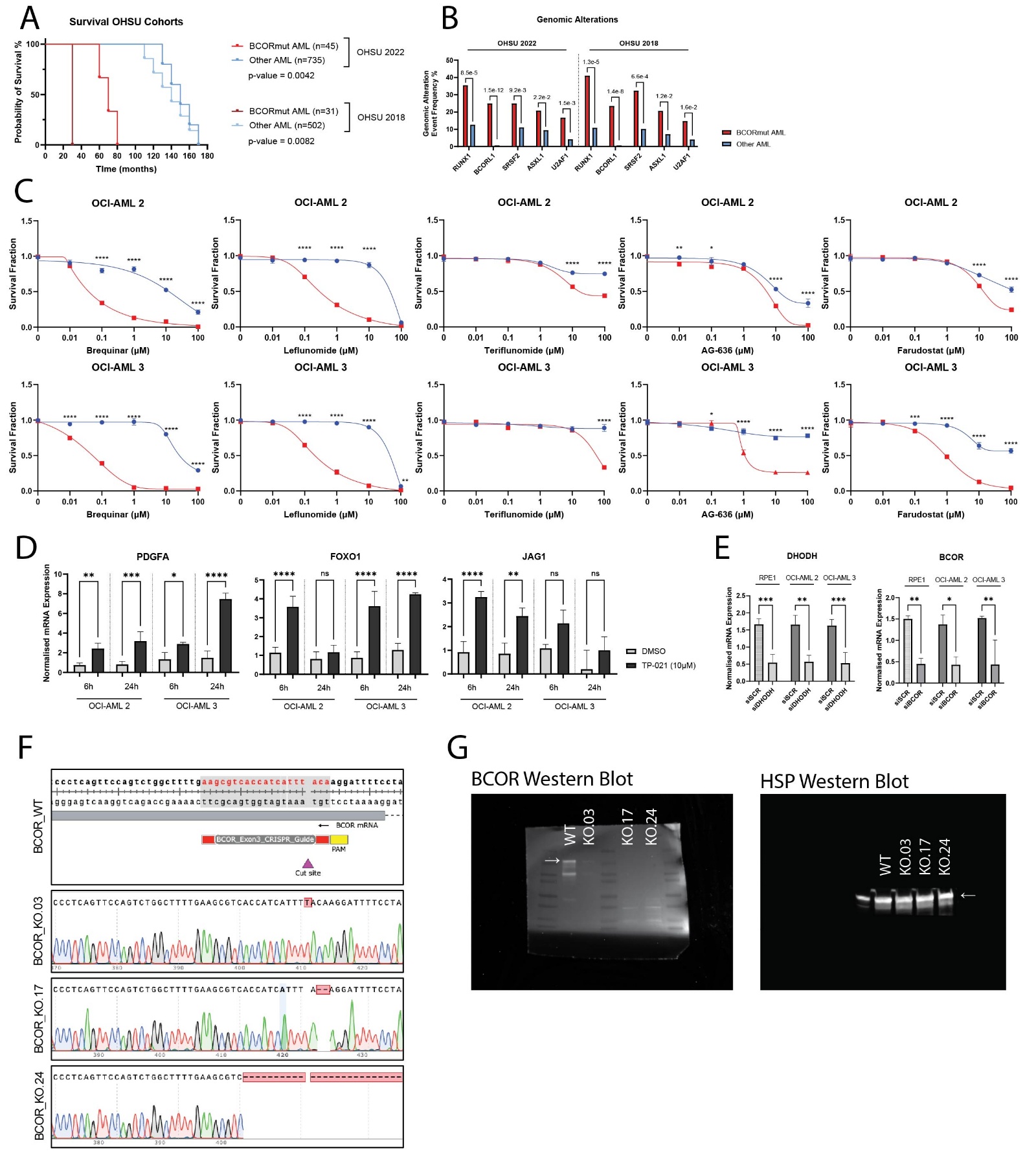
^

***Figure S.1:*** *(A) Survival curves comparing BCOR-mutant AML to Other AMLs, in both OHSU cohort from 2022 (Cancer Cell) and 2018 (Nature). Data were collected on CBioPortal.org. P-value are calculated using log-rank (Mantel-Cox) test. (B) Genomic alterations of 5 consensus genes found across the two cohorts, with higher frequencies in BCOR-mutant AMLs. P-value are calculated using two-side Fisher Exact test. (C) mRNA Expression of FOXO1, PDGFA and JAG1 normalised to GAPDH Expression. OCI-AML 2 and OCI-AML 3 were treated whether with TP-021 (10µM) or DMSO for 6 or 24hrs (one-way ANOVA, n=6; p<0.0001****; p<0.001***; p<0.01**; p<0.05*). (D) Survival curves of OCI-AML 2 and OCI-AML 3 treated with increasing doses of DHODH inhibitors ± TP-021 (10µM) (two-way ANOVA, n=3; p<0.0001****, p<0.001***, p<0.01**, p<0.05*). (E) mRNA Expression of BCOR and DHODH confirming effective knock-down in transfected cells (one-way ANOVA, n=3; p<0.001***, p<0.01**, p<0.05*). (F) SnapGene snapshot of Sanger sequencing data for BCOR exon 3 in OCI-AML3^BCOR_WT^ and newly generated BCOR-mutant: OCI-AML3^BCOR_KO.03^, OCI-AML3^BCOR_KO.17^ and OCI-AML3^BCOR_KO.24^. OCI-AML3^BCOR_KO.03^, OCI-AML3^BCOR_KO.17^ contain a T insertion and a CA deletion, respectively, both of which lead to the insertion of a stop codon. OCI-AML3^BCOR_KO.24^ exhibits a large deletion in exon 3. (G) Western Blot (WB) characterisation of single-cell subcloned OCI-AML3^BCOR_WT^, OCI-AML3^BCOR_KO.03^, OCI-AML3^BCOR_KO.17^ and OCI-AML3^BCOR_KO.24^.*

**
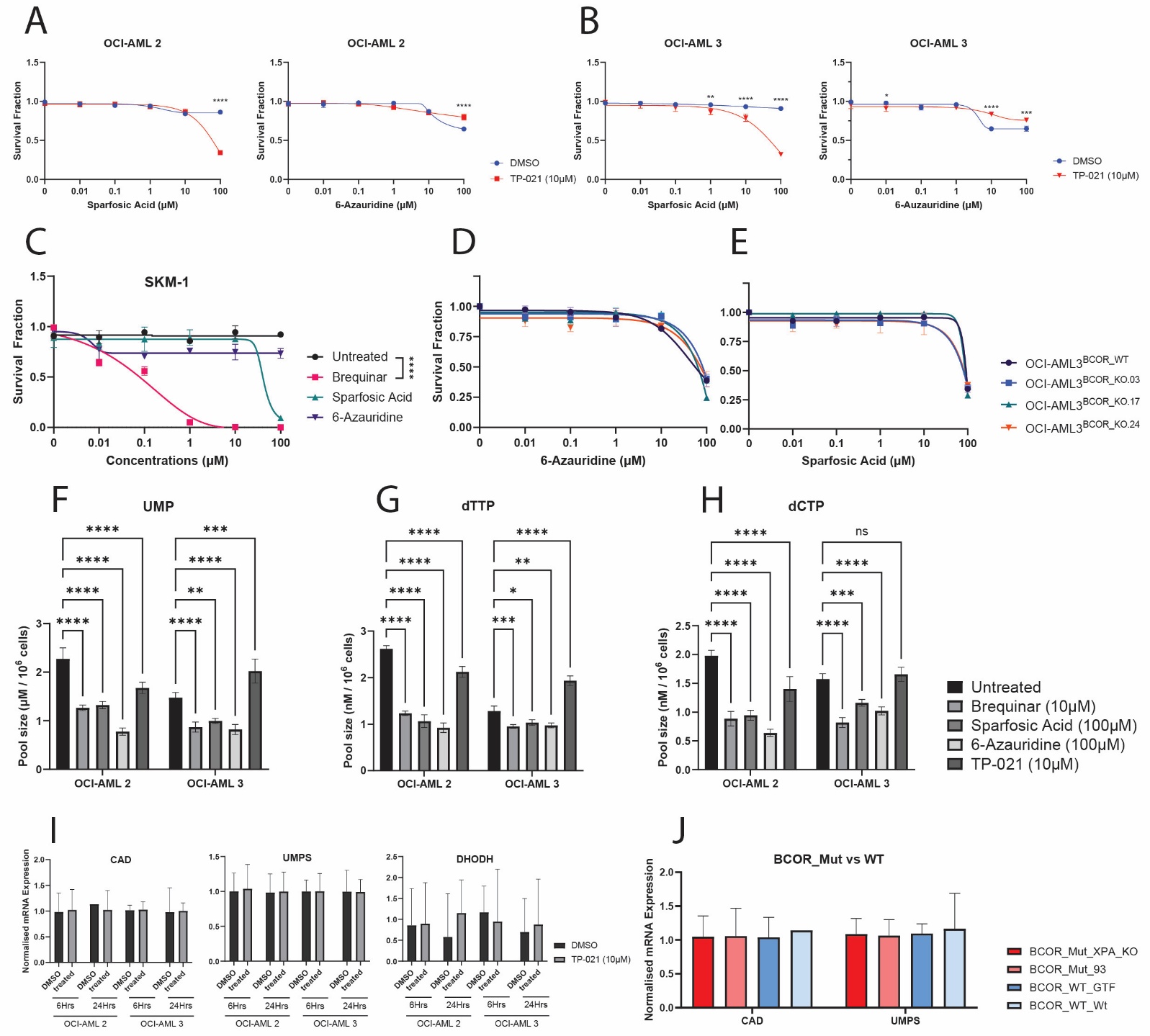
**

***Figure S.2: DHODH dependency of BCOR-mutant cells is not due to its role in the de novo Pyrimidine pathway****. We investigated whether the vulnerability of BCOR-deficient cells to DHODH inhibition reflected a dependency on the de novo pyrimidine pathway. Inhibition of CAD (upstream of DHODH) or UMPS (downstream) produced little to no effect on viability, even in BCOR-deficient cells, in contrast to the strong impact of DHODH inhibition. Consistently, while CAD, UMPS, and DHODH inhibition each reduced UMP and downstream nucleotides (dTTP, dCTP), BCOR inhibition itself did not alter nucleotide pools. Gene expression of pyrimidine biosynthesis enzymes (CAD, DHODH, UMPS) was also unchanged upon BCOR inhibition or loss. Together, these results indicate that BCOR deficiency does not impose a greater demand on pyrimidine synthesis, and that the heightened sensitivity to DHODH inhibition more likely reflects an alternative function of DHODH beyond its canonical role in nucleotide biosynthesis.*

*Survival curves of (A) OCI-AML2 and (B) OCI-AML3 treated with increasing doses of sparfosic acid (CAD inhibitor) or 6-azauridine (UMPS inhibitor) ± TP-021 (10µM). (C) Survival curves of SKM-1 cells treated with increasing doses of CAD or UMPS inhibitors. Survival curves of OCI-AML3^BCOR_WT^, OCI-AML3^BCOR_KO.03^, OCI-AML3^BCOR_KO.17^ and OCI-AML3^BCOR_KO.24^ treated with increasing doses of CAD or UMPS inhibitors (D, E). OCI-AML2/OCI-AML3 were treated 24Hrs with brequinar, sparfosic acid, 6-azauridine or TP-021 to quantify (F) UMP, (G) dTTP and (H) dCTP levels (two-way ANOVA, n=3; p<0.0001****, p<0.001***, p<0.01**, p<0.05*).* *(I) CAD, DHODH and UMPS gene expression were measured in OCI-AML 2, -3 ± TP-021 (10µM), treatments were applied for 6 or 24hrs. (J) CAD and UMPS gene expression were measured in 2 BCOR-mutant and 2 BCOR-wildtype hiPSC cell lines, obtained from Xeroderma Pigmentosum patients.(two-way ANOVA; n=5; p=n.s).*

**
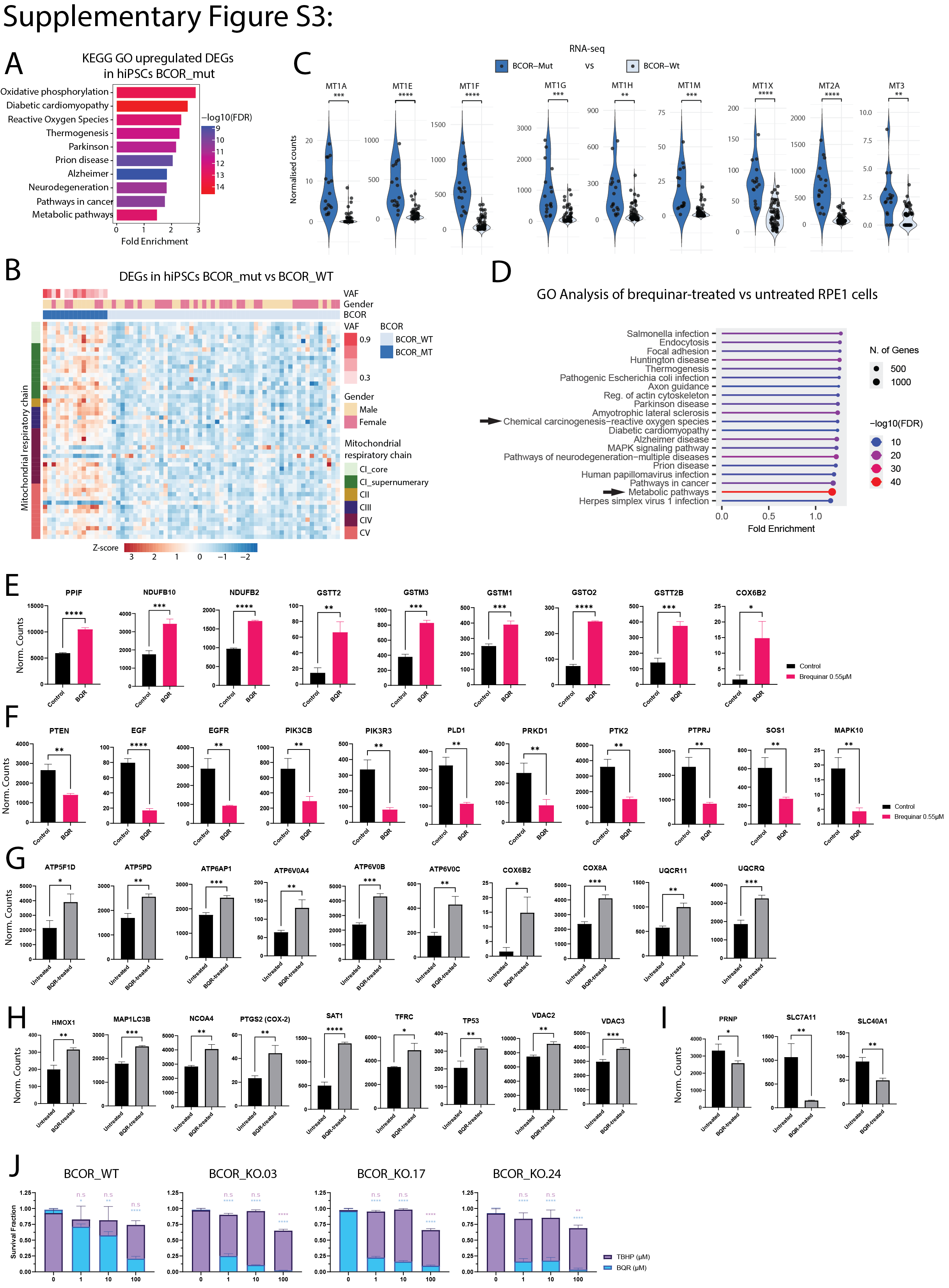
**

***Figure S.3:*** *(A)* *Gene Ontology of upregulated Differentially Expressed Genes (DEGs) in BCOR-mutant contrasted to BCOR-wildtype hiPSCs, showing significant upregulation of Oxidative Phosphorylation, Reactive Oxygen Species and Metabolic Pathways indicating BCOR-mutant cells live in higher oxidative stress environment. (B) Heatmap of DEGs involved in mitochondrial electron transport chain (ETC) in BCOR-mutant and Wildtype hiPSCs. This significant increase of ETC-involved gene expression in BCOR-mutant cells indicate higher mitochondrial metabolism and potentially higher oxidative stress. (C) Normalized RNAseq counts of Metallothionein genes in BCOR- mutant compared to wildtype hiPSCs. Metallothionein proteins are known to be ROS scavengers, where significantly increased expression indicates high ROS levels (one-way ANOVA, n=16 for BCOR-mutant and n=43 for wildtype; p<0.0001****, p<0.001***, p<0.01**). (D) Gene Ontology analysis of top significantly differentially expressed genes (DEGs) contrasting RPE1 cells treated with brequinar (0.55µM, 24Hrs) versus untreated, showing ROS and Metabolic Pathways as top enriched pathway in brequinar-treated cells. Normalised counts of mRNA expression of enriched genes involved in (E) ROS pathway and (F) EGF/HFG pathway, observed in the volcano plot from figure 3. (two-way ANOVA, n=3; p<0.0001****, p<0.001***, p<0.01**, p<0.05*). (G) Normalised RNAseq counts of RPE1 untreated and BQR-treated, showing Brequinar treatment at 0.55µM for 24hrs induces expression of genes involved in Oxidative Phosphorylation (two-way ANOVA, n=3; p<0.0001****, p<0.001***, p<0.01**, p<0.05*). (H) Normalised RNAseq counts of RPE1 untreated and BQR-treated, showing Brequinar treatment at 0.55µM for 24hrs induces expression of genes involved in Ferroptosis triggering (two-way ANOVA, n=3; p<0.0001****, p<0.001***, p<0.01**, p<0.05*). (I) Normalised RNAseq counts of RPE1 untreated and BQR-treated, showing Brequinar treatment at 0.55µM for 24hrs represses expression of genes involved in Ferroptosis protection (two-way ANOVA, n=3; p<0.0001****, p<0.001***, p<0.01**, p<0.05*). (J) High doses of TBHP, a ROS inducer, fails to induce cell death in OCI-AML3^BCOR_WT^, OCI-AML3^BCOR_KO.03^, OCI-AML3^BCOR_KO.17^ and OCI-AML3^BCOR_KO.24^ after 6 days of treatment, showing the specific importance of DHODH in maintaining efficient mitochondrial metabolism for survival in high ROS environment (two-way ANOVA, n=3; p<0.0001****, p<0.001**, p<0.05*, n.s = non-significant – All compared to untreated condition).*

**
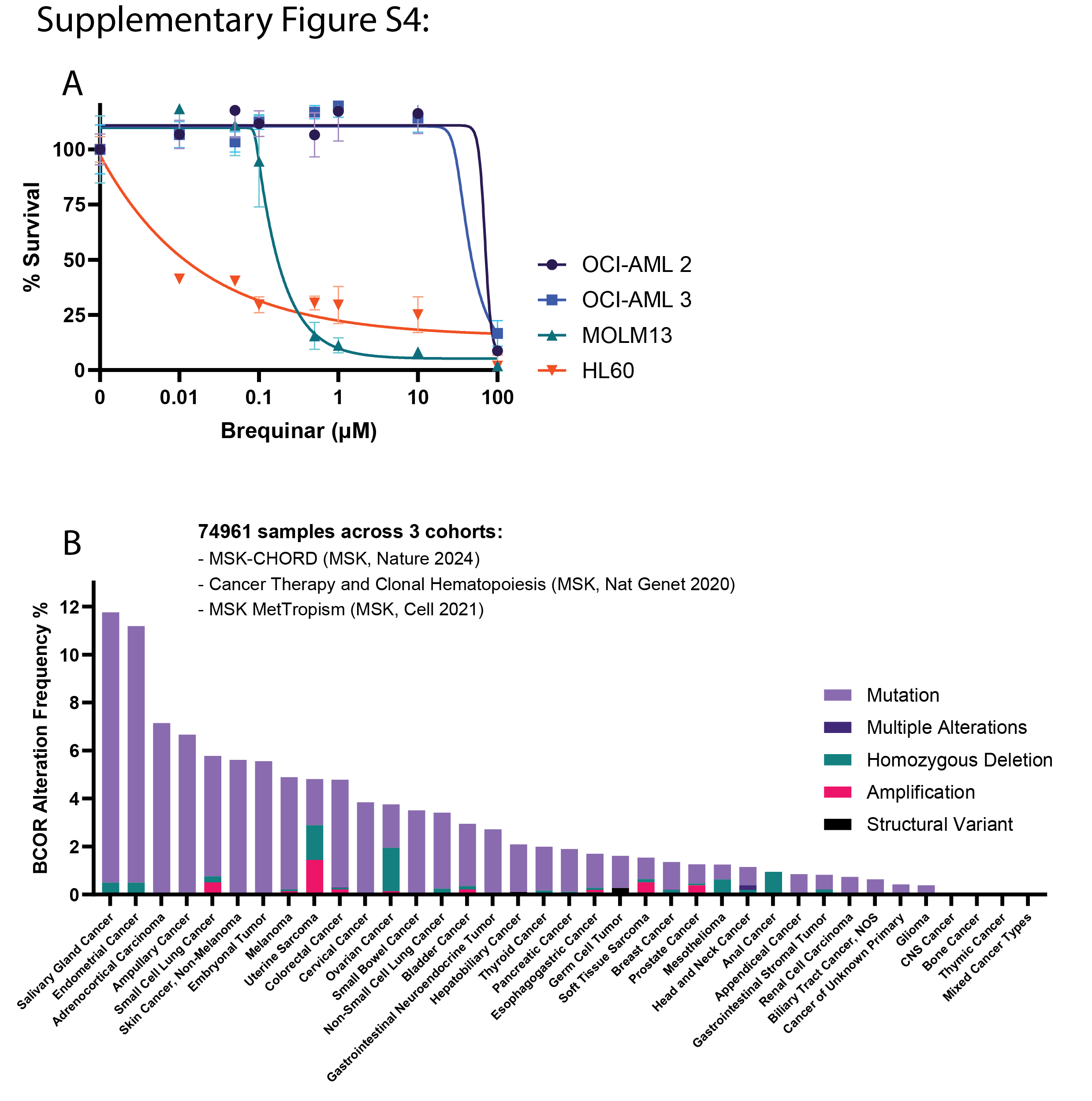
**

***Figure S.4:*** *(A) OCI-AML2, -3, MOLM13 and HL60 were treated with increasing doses of Brequinar for 6 days, and cytotoxicity was evaluated with Promega Cell-Titer Glo. (two-way Anova: MOLM13 vs OCI-AML 2/3: p<0.0001**** from 0.5 to 10µM; HL60 vs OCI-AML 2/3: p<0.0001**** from 0.01 to 10µM). (B) BCOR Alteration Frequency observed in different types of Cancer, analysis of 74961 samples over 3 cohorts (MSK-CHORD, Cancer Therapy and Clonal Hematopoiesis, MSK MetTropism), data collected on CBioPortal.org.*
